# Clinical Review, and Hospital Reports

**Published:** 1841-04-01

**Authors:** 


### Clinical Review.

### ROYAL INFIRMARY OF EDINBURGH.

Cases in Surgery from the Clinical Practice of Professor Syme. Reported by J. Henderson Hardie, M.D. Edinburgh.*

1. *Injuries of the Head with Concussion of the Brain—Bleeding from the Ear—Recovery.*

*Case* 5.—John Macleod, aged twenty-four, was thrown out of his cart on the 23d of March, and sustained a compound fracture of the frontal bone, there being merely a fissure with slight depression on one side. On admission he was insensible, and had profuse bleeding from the right ear. He gradually recovered without operation, deafness on the right side being the only permanent inconvenience resulting from the injury, though an inequality on the surface of the bone can still be perceived.

*Case* 6.—Joseph Ross, aged thirty-four, had fallen into a dry dock at Leith, on the 10th of August last, when he was found labouring under the symptoms of concussion of the brain, with considerable bleeding from the left ear. The ordinary treatment for such injuries was pursued, and he was dismissed six weeks afterwards, complaining only of deafness on the left side.

*Case* 7.—John Thomson, aged twenty-six, was admitted on the 5th of September, having just received a severe blow on the head and left shoulder by the fall of a mass of iron, weighing twelve hundred weight, which, fortunately, only grazed those parts in its descent.

The consequence was fracture through the body of the scapula, and an injury at the base of the skull which occasioned copious hæmorrhage from the left ear. He quitted the hospital on the 12th of October, able to resume his employment as a labourer, and suffering only from deafness on the left side.

The reporter observes—as bleeding from the ear is by some considered a fatal symptom when attending concussion of the brain, it has been thought right to mention the preceding cases of recovery under such circumstances.

The hæmorrhage no doubt denotes a fissure at the base of the skull, extending through the lateral sinus, certainly a very dangerous occurrence, though not necessarily a mortal one.

The reporter mentions the following curious case:—

In the spring of last year Dr. James Wood asked Mr. Syme to see a young gentleman, eleven years of age, on account of an alarming hæmorrhage from his ear. He was recovering from a severe attack of scarlatina, in consequence of which both ears had suppurated, when, upon the fifteenth day, a large quantity of blood was suddenly discharged from the right side. During the six succeeding days the bleeding returned three times, to the extent, by computation, of a pound on each occasion. It was deemed proper to place a ligature on the carotid artery, which was concluded to be the source of the hæmorrhage. Bleeding recurred while the operation was being performed; and twice again, to a small extent, not exceeding a few tea-spoonfuls, in the course of the following evening and night.

For several days afterwards there was hardly any appearance of blood, and all the circumstances encouraged the entertainment of favourable hopes. Symp-

* Edinburgh Monthly Journal of Medical Science.

toms of cerebral excitement, however, then showed themselves, and terminated fatally on the eleventh day after the operation.

On examination it was found that the carotid artery was not concerned in the disease; but that a small ulcerated aperture, in the osseous septum between the termination of the lateral sinus and the cavity of the ear, had permitted the blood to escape from this vessel.

Could this have been ascertained previously, stuffing the ear would, of course, have suggested itself as the proper practice.

2. *Punctured Fracture of the Skull—Wound of the Sinus—Operation—Death.*

Biddie Drummond, aged seven years, was admitted on the 28th of February last, having just received an injury on the back of her head from a stroke with a large stone. She became insensible immediately after the accident, and on admission was very drowsy and confused, with stertorous breathing, an intermittent pulse, and a cold surface. Her head was shaved and warmth was applied to her body generally, till Mr. Syme's arrival two hours after she was received into the hospital. A punctured fracture being found—at the occiput in the mesial line—it was considered necessary to apply the trepan in order to remove the depressed bone. This being done, a loose scale of the internal table was removed, immediately after which the child became conscious and answered questions; but a profuse gush of dark-coloured blood issued from the bottom of the wound, and came from the superior longitudinal sinus, opened by the bony spiculum. This hæmorrhage was suppressed by means of a compress and bandage.

Everything went on favourably—the wound and dura mater looking well, the child sitting up in bed—until a week after the operation, when, during a fit of coughing, at least a tumbler-full of blood issued from the wound, inducing syncope; a dossil of lint was placed in the opening, and over it a piece of cork, firmly retained by a bandage. This had the effect of stopping the hæmorrhage for the time.

Two days afterwards considerable restlessness came on, with profuse perspiration, intermitting pulse, insensibility, and other signs of sinking. She died in the course of the night. On examining her head the brain and membranes had an exsanguine appearance, and five drachms of serum were found in the lateral ventricles. An opening, much larger than that seen during the operation, existed in the superior longitudinal sinus at the seat of injury; the margin of this aperture was sloughy, ulcerated, and covered with pus. A fibrinous clot lay in the sinus for some distance anterior to the opening in it, but did not, in any way, tend to close the breach in this vessel.

3. *Gunshot Wound of the Skull—Operation—Hernia Cerebri—Death.*

Robert Blake, aged twenty-seven, was admitted on the 1st of July, after having walked from Habbieshow, at the Pentland Hills, a distance of about eight miles from town. He stated that, from the accidental discharge of a pistol, a bullet was lodged in his head, behind the right ear.

On examination it was found to be so; but from other information, and more particularly from his having attempted suicide some time before, it was thought that the injury had been inflicted intentionally.

The wound—an inch and a half above and behind the right ear—was small and circular, with inverted, blackened edges. A bullet was found firmly impacted in the skull at the bottom of the wound; this being extracted, it appeared that the subjacent bone was depressed. The patient was excited, without any marks of compression.

The trepan was applied by Mr. Syme over the seat of depression, and seven loose fragments of both tables removed. A small clot of blood lay on the dura mater, which appeared rather torn and uneven. Cold applications were ordered to the head, and strict rest was enjoined. In the evening the patient was bled to syncope, and a large dose of colocynth and calomel was administered. On the following day he complained of headache; his pulse rose rapidly, from 80 to above 100, and he became restless; he was bled to fourteen ounces, and the same purge was repeated. On the 3d he felt much pain in the forehead; to alleviate which, twelve leeches were applied to this part; and in the evening—in consequence of aggravation of the pain—sixteen ounces more of blood were removed from the arm, and a purge—consisting of colocynth, gr. xv. and croton oil gtt. i.—was given. A poultice was then applied to the wound, at the bottom of which the dura mater was seen discoloured. On the 8th, a soft round swelling, as large as a nutmeg, presented itself at the opening in the skull; a bandage and a compress were applied in order to oppose its increase. On the 9th another protrusion manifested itself at the side of that just mentioned; his pulse being rather full, he was bled to the entent of ten ounces. On the 10th, the protruded part was shaved off to a level with the opening in the cranium; and compresses with a bandage were applied. The portion removed weighed 110 grains, and, though in a sloughy state, showed traces of the cerebral substance mixed with blood. Another protrusion presented itself on the 12th; it, however, seemed to be a mere slough.

At this time the patient had a severe rigor; he was feverish, and laboured under insensibility, whilst the signs of approaching sinking rapidly made their appearance. He died on the 17th of July. The head was examined after death, when the dura mater, at the seat of injury, was seen perforated, sloughy, and discoloured; at the same part an abscess, about as large as a walnut, existed in the substance of the brain, which was otherwise in a healthy condition.

### 4. *Dislocation of the Humerus into the Axilla of seven weeks' standing—Reduction.*

William Stewart, aged fifty-six, a slater from Dumfermline, fell on his right shoulder seven weeks before his admission into the Hospital, on the 3d of December last. The case was looked upon by a bone-doctor in the country as a bruise, and was treated accordingly. All the characters of dislocation of the humerus inwards, however, were well marked, the bone was rather farther forward than usual, and the abnormal direction it followed was particularly observable. After having been for an hour in the warm bath, he was placed horizontally, and subjected to extension, by means of the pulleys, in the direction of the long axis of the body. The bone regained its place without any snap, but escaped on the extension being discontinued. Again reduced, it was retained *in situ* by a spica bandage, and a pad in the axilla.

In this case it was supposed that the glenoid cavity of the scapula had either been altered in its form by absorption, or had been fractured at the time of the accident, and that, in consquence, the head of the humerus was permitted to leave its place.

The warm bath was used in the case just related, as considerable difficulty was anticipated from the long standing of the injury. In such cases Professor Syme generally has recourse to this means, occasionally aided by blood-letting, and nausea kept up by repeated small doses of the tartarized antimony, given previously to the attempt, as it is found difficult to induce this state whilst the efforts at reduction are being made.

"Besides the above case of dislocated shoulder, three recent luxations of this joint presented themselves; they were all into the axilla. In the first of these I effected reduction by means of the heel in the axilla whilst extension was made from the wrist; in the second by placing the knee in the axilla whilst the arm was extended transversely; and in the third case, which had just happened, I attained the object, without any assistance, by suddenly abducting the arm, rotating it outwards, and thrusting the bone forwards.

In the first of these three cases the method mentioned was followed, as some difficulty was anticipated, and assistance could not be readily procured. The second method seems, in ordinary circumstances, the most convenient;—whilst the one last mentioned has been found generally to answer, when an opportunity was afforded of effecting reduction within two or three hours after the injury had been received."

5. *Stricture of the Urethra—Incision—Cure.*

Alexander Williamson, aged twenty-four, from Queensferry, was admitted on the 24th of August last, with a very tight stricture of the urethra between two or three inches from its orifice. About two months previously he had retention of urine, and applied to a practitioner for relief. Attempts were made to introduce the catheter without success, but with the effect of causing great pain, and a copious flow of blood. Mr. Syme tried repeatedly, and on different days, but in vain, to pass a catheter of the smallest size, flexible as well as solid; and at length resolved to cut through the strictured part. This was done by introducing the point of a sharp-pointed curved bistoury through the integuments, upon the extremity of a bougie pressed down as far as possible along the canal.

A full-sized catheter was immediately afterwards passed into the bladder, and retained there for twenty-four hours. The wound was quite healed on the fourth day, when the man left the hospital. No. 11 of the catheter scale has been passed several times since then, and the canal does not evince any disposition whatever to contract.

It is remarked, that when strictures occur in the anterior part of the urethra, they are apt to acquire an extreme degree of tightness. If situated at a greater distance from the orifice, their apparent narrowness may be attributed to the difficulty of guiding instruments through them. But the superficial position of those now under consideration, prevents any such ambiguity, and the successful issue of the case just related, affords encouragement to employ the summary measure of incision, when the ordinary method of dilatation proves impracticable.

6. *Hare Lip.*

Some cases are related for the purpose of shewing,—

1st. The impropriety of operating at an early age. 2d. The advantage,—both as regards the firmness of union, and the appearance of the lip,—derived from a free removal of the round and thickened margins of the fissure. 3d. The danger of leaving the lip unsupported sooner than the fourth day, at least, after the operation.

7. *Popliteal Aneurysm.*

1. *Spontaneous Cure.*—Henry Williams, aged thirty-six, a weaver, was admitted on the 20th May, 1839, on the recommendation of Mr. Cunningham of Kirkcaldy, to have the femoral artery tied for popliteal aneurysm. The tumor occupied the hollow of the ham—it was circumscribed in form—and, from the distinctness of its pulsation, seemed to contain little coagulum. The patient's attention had been first directed to the complaint about two months before, by an uneasy feeling of stiffness in the part, after a particularly severe day's work.

He was confined to bed, and ordered a laxative to prepare him for the operation. Next day the pulsation had become extremely obscure, and though it slightly returned the following day, at the end of two days more it could not be perceived at all. The articular arteries were then felt to be much enlarged, and the tumor quickly diminished in size, while it increased in firmness, until merely a small knot the size of an olive remained. He was dismissed at his own desire on the 31st of May.

It is not very often that aneurysm in an early stage is cured spontaneously. Mr. Syme says,—"It has been a question whether an early or advanced stage of the disease is more favourable for success,—the undilated state of the anastomosing vessels being considered adverse in the former, and the quantity of extravasated blood an obstacle in the latter. From all that has fallen within my own observation, I should have no hesitation in preferring to operate at an early period, having never witnessed in my own practice the slightest unpleasant symptom of defective circulation, however small and recent the tumor might be."

Mr. Syme comments on the bad consequences that ensue from ligature of the femoral artery. He has, however, tied it seven times with success. "But within the period of doing so, I am not aware of any case that has terminated favourably in this city, while I have either seen or heard of four that ended badly, viz. one by inflammation of the vein, one by mortification, one by hæmorrhage, and one by amputation." He observes that the femoral artery has a closer connexion with the vein, and though it is felt by the operator's finger, after the fascia has been opened, round and distinct, and as if insulated from the surrounding parts, except by the loosest connexions, any attempt to pass the ligature, without further dissection, either proves abortive, or if executed by force, exposes the patient to the greatest danger. "I have seen a gush of dark-coloured blood proclaim transfixion of the vein; I have seen on dissection a portion of this vessel included in the ligature; and I have also seen the external coat alone grazed, as it were, by the needle, but nevertheless excited to fatal inflammation. If, on the other hand, this danger be avoided by using blunt instruments, or the finger to detach the artery from its connexions, the patient is exposed to the hardly less disastrous consequence of hæmorrhage, through ulceration or sloughing of the vessel.

To tie the femoral artery safely, the surgeon should be impressed with the conviction that the operation is one not of difficulty, but of great nicety. He should make an incision between two and a half and three inches long in the proper situation, cut through the fascia to a smaller extent, and expose the sheath of the vessels. So far he can hardly go wrong; but then, instead of hastening to pass his needle, he should, by ligature, or the temporary application of spring forceps, close every little vessel that discharges enough of blood to obscure distinct vision of the object he has in view. Let him now seize the sheath with dissecting forceps, and gently raising it, make a small opening by means of a straight narrow sharp-pointed knife. The cellular and fatty substances which envelope the vessels in variable quantity, are next to be elevated and divided in successive portions, until the external coat of the artery appears quite distinct and *white*, when the needle may be passed without the slightest difficulty or danger."

### 8. *Brachial Aneurysm.—Sac laid open.*

*Case.*—William Smith, aged twenty-three, was admitted on the 19th of October last, on account or a pulsating tumor at the bend of the arm, which had resulted from his being bled there nine weeks before. In consequence of the pressure that had been used to remedy the aneurysm, the skin covering it was ulcerated to a small extent; the pulse at the wrist was nearly as strong as in the sound limb.

On the following day, Mr. S. laid open the sac, having previously applied a tourniquet, turned out the clots, and readily discovering the wound of the artery, passed the needle first above and then below it, so as to convey a couple of ligatures, which were tightly tied. No bad symptoms followed, and the patient was dismissed on the 18th of November quite well.

Mr. Syme always resorts to this operation which he finds as effectual as easy.

9. *Varicose Brachial Aneurysm—Operation.*

*Case.*—Agnes Easton, aged twenty-three, admitted May 28, on account of injury in venesection three months before. On examination, it appeared that a communication between the artery and vein still existed, through the medium of the aneurysm, the latter of the vessels being considerably distended, and conveying a jarring sensation to the hand placed over it, while the characteristic purring sound was distinctly heard, by applying the ear, either directly or with the intervention of the stethoscope.

A tourniquet having been screwed on, the sac was laid open freely, so as to avoid the vein. Instead of the laminated coagulum, which lines the interior of aneurysms, presenting itself, it was then seen that the cavity contained only fluid blood, and that the surface of its parietes was perfectly smooth, white, and, in short, similar to that of an artery. There was hence some difficulty in detecting the wounded part of the vessel; and it was necessary, partly by dissection, but chiefly by tearing, to remove the principal part of the sac. The orifice being then discovered, Mr. S. exposed the artery above and below it, so as to pass a ligature at each of these points. No inconvenience was experienced, pulsation being felt at the wrist the evening after the operation, and the patient was dismissed quite well on the 16th of June.

The patency of the communication with the vein, in this instance, prevented the remora and coagulation of blood in the tumor; and ligature of the artery, without opening the latter, would probably have been of little service.

10. *Aneurysm by Anastomosis—Excision.*

" William Farquharson, aged fourteen, from Perthshire, was admitted on the 7th of September, 1839.

Having finished my visit, I was about to leave the hospital, when the house-surgeon told me that a case had just been admitted under my care, that required immediate attention. The complaint, he said, was a sore on the leg which had a great disposition to bleed, and that having taken off a bandage from the limb, he had found it necessary to apply a tourniquet, to arrest the profuse hæmorrhage which ensued. On going into the ward, I found the patient in bed, with his leg lying in a pool of blood. The outer side of it, a little below the knee, presented a discoloured and slightly elevated surface, extending from the head of the *fibula* about four inches and a half downwards, and three in breadth. The blood appeared to have issued from two small irregular ulcerated openings near the centre of this part. Over these compresses were placed, and secured by a roller applied firmly from the toe upwards. This arrested the hæmorrhage, and afforded time to inquire into the case, and consider what should be done.

The patient stated that, to the furthest extent of his recollection, there had been a dark-coloured mark, about the size of a half-crown piece, in the seat of the disease; but that this had occasioned no inconvenience until two years before, when an opening took place, and blood, in large quantity, gushed out. The discolouration had then extended, and been accompanied with elevation of the surface. Subsequently the bleeding frequently returned, and in his lonely occupation of a shepherd, repeatedly threatened to prove fatal. Latterly he had suffered in this way every two or three weeks, and become so much exhausted, that his friends, seeing him about to sink under the complaint, undertook the long journey from their distant residence in the Highlands, in quest of relief.

There could be no doubt as to the nature of the disease, which was evidently a tumor composed of erectile texture; but great difficulty seemed to lie in the way of any efficient treatment. The large superficial extent and position in part over the bone, rendered the use of ligatures inapplicable, while from the uncertain depth, and large portion of skin engaged, excision appeared an operation not only formidable in its execution, but unpromising in its result. In these circumstances, I resolved to attempt the excitement of inflammation and adhesion between the intersticial surfaces of the morbid structure, and with this view, two days afterwards, having previously applied a tourniquet, cut through the whole length of the discoloured integuments, detached them from their subjacent connexions, and stuffed the cavity with lint, the bandage being then applied as before.

Every thing went on well for six days, when the roller being loosened, blood streamed out with undiminished force. The patient was now reduced to a state of extreme weakness, and I was in despair of relieving him in any other way than by amputation, when a gentleman, whose valued acquaintance I owe to this case—Dr. Little of Sligo, encouraged me to try extirpation of the disease, by mentioning that he had known this done with success on an equally unpromising occasion. The whole of the unsound surface having been embraced by an elliptical incision, I divided the fascia, and was glad to find the muscular substance not implicated. Cutting through it parallel to the base of the disease, I arrived at the bone, and readily completed the separation from it, as the periosteum was not engaged. Three or four vessels were then tied, and lint being applied to restrain any oozing from the surface, a roller was put on, and the tourniquet removed. No unpleasant symptom followed, and though the cure, as might be expected, proved tedious, it was ultimately complete."

---

### GUY'S HOSPITAL.

### 1. *Fatal Case of Chorea.**

The patient was a sickly-looking boy, aged fourteen. He appears to have enjoyed good health until he was five years of age, when he received a blow on the vertex of the head from a shovel, which caused a deep wound of about two inches in extent. During the process of healing, pains arose in the head, and have continued almost without any intermission to disturb him ever since. About a month after the injury, his mother, for the first time, observed that he was affected with convulsions. The attack then assumed a very severe form, both as to intensity and duration, and similar attacks have, from that time, constantly recurred, but at irregular periods, the intervals of the disease gradually lengthening, while the disease itself became less and less severe at each successive time of its occurrence. Three months ago, however, his sister died, and the news of her decease having been told to him somewhat suddenly, affected him very much. He rapidly became much worse in health; his headache assumed a very aggravated form, and he complained of weakness in his left side. Five days previously to his admission into the hospital, to all appearance without any immediately exciting cause, he was seized with an attack of chorea of a most violent form, so that at one time it required four men to hold him. He screamed loudly at night, foamed at the mouth, and attempted to bite those who approached him. The attack was ushered in by increased intensity of headache, and by vomiting, which last symptom continued for three days. He rapidly became worse; could not obtain rest nor swallow food, and lost all power of articulation. When admitted into the ward he appeared much worn, and his convulsive actions were so violent that it was necessary to strap him down to his bed. The treatment consisted in the administration of hyoscyamus and camphor at night, and ammonia in the day. Under this he appeared to

---

* Prov. Med. and Surg. Journ. Dec. 5, 1840.

improve, for the convulsions and cephalalgia became less and less severe, and his articulation to a great extent returned; but notwithstanding support, as beef tea and wine, were given, in a few days he gradually sank, exhausted. Previous to his admission he had been bled, a measure which Dr. Bright considered very seldom judicious, excepting when the disease occurs in robust ruddy young women. It is curious that, notwithstanding the most careful examination after death, nothing morbid was detected either in the brain or spinal cord. There was a slight inflammatory deposit on the dura mater, but not corresponding with the site of the original blow, and certainly altogether insufficient to cause the disease.

2. *Mr. Bransby Cooper upon Hydrocele.**

*Abnormal Position of the Testis.*—Do not merely satisfy yourselves as to the existence of a transparent tumor, and proceed at once to puncture it; but having the room darkened, and the light shaded by your hand, examine if the opacity denoting the situation of the testicle is behind, as is usually the case, before, or at one side. I have had to puncture at the back of the sac, because the testicle was adherent to the anterior part; but if practicable, it is always better to do it on the side, because as may be seen in this preparation, a number of adhesive bands may cross the cavity of the tunica vaginalis, uniting the testicle to the anterior part of the sac, but leaving the cord behind, and in the latter case, you might wound the spermatic artery by a posterior puncture. Sometimes these adhesions of organized lymph will form two or three sacs, constituting what is called encysted hydrocele; and then when you tap, instead of getting ten or twelve ounces of fluid, as you expected from the size of the tumor, you find only two or three. In this case each cyst may be opened by separate punctures through the scrotum; or you may do as I have been obliged to do, evacuate the fluid by passing a needle along the canula without this having been withdrawn. You should never inject in these cases, because the existence of the cysts depends on inflammation, and evidences such a tendency to inflammatory action, that the mere puncture will probably produce a radical cure. Injection, perhaps, would be admissible, however, after a second tapping. Sometimes you have only one of these cysts, and that a very small one

*Case.*—A gentleman, aged 54, consulted me respecting a disease of the left testicle, from which, however, he suffered so little, as at times to lead him to suppose the disease was imaginary, although a small swelling was perceptible, which I examined and found at the upper part of the testicle, just over the head of the epididymis. It was soft and fluctuating; but at first I merely suspected it was a distension of the spermatic veins,—in fact, a partial varicocele. Being doubtful, however, I examined it with a candle, when the nature of the disease was rendered clear, by its perfect transparency. The tumor being tender to the touch, and so small and deeply seated, I ordered a few leeches, evaporating lotions, purging, and the use of a suspensory bandage, lined with oil-silk, rather than puncture in the inflamed state; and I believe the gentleman quite recovered.

Now, I am not quite sure that this was a cyst of the tunica vaginalis, as it might have been merely an adventitious formation, external to the serous membane. I know of no way of distinguishing this before puncture, but the nature of the fluid will determine it; for if the fluid have been secreted from the tunica vaginalis, it will be found highly albuminous, coagulating by heat and nitric acid, while the fluid of the more superficial cysts contains a very slight trace of albumen.

---

* Prov. Med. and Surg. Journ. Jan. 30, and Feb. 6, 1841.

*Complication with Hernia and in Old People.*—There is another point you must ascertain before operating, and that is, whether the hydrocele be complicated with hernia. Your patient states that he had the usual well-known symptom of hernia, and you find the posterior portion of the tumor opaque. Well, even if the hernia be irreducible, there is no danger in the palliative treatment, if you take due caution; but it is better not to inject, for fear of inflammation extending to the hernial sac. You would also be satisfied with the palliative means in very old people, whose constitutions it is found are incapable of supporting acute local inflammation; and you will sometimes be called to operate on persons of a very advanced age. I remember an old Norfolk admiral coming to me with a very large hydrocele, and he was either 89 or 90 years of age; so, before puncturing, I asked him if it gave him much pain. "Oh! no," said he, "but Mrs. H. finds it very inconvenient." (loud laughter.) Neither should I inject a hydrocele, the first time of tapping; for it often happens, though the patient may have complained of very little pain, that the mere irritation of the canula sets up an altered action sufficient to effect a permanent cure.

*Iodine Injection.*—For the last two or three years Mr. Cooper has used this exclusively—two drachms of tincture of iodine and six drachms of water; two drachms of which mixture may be injected and left. I have a note here stating that Dr. Henry Goodeve has used this injection in India in 276 cases, and has only failed twice. Since I have used the iodine, I have not had a single case where the injection failed to effect the radical cure, and only one where I had to repeat the injection. This was a case where the tincture of iodine that I used was bad, and as I felt convinced it would not succeed, I repeated the injection four days afterwards with some which had been properly prepared. The chemists will tell you more of this matter than I can; but if the tincture be made without the admixture of hydriodate of potash, a precipitate forms when you add the water, and the mixture is quite useless, but when properly made the watery solution is perfectly bright.

*Always place your Patient in a chair.*—Always place your patient on a sofa, or let him lean back in a chair, because any one is liable to faint, however bold he may appear; and then, if you have operated while he is erect, he falls, probably upsets the basin, hurts himself, or frightens his friends, all which appear very clumsy, especially in a private house.

*Never let a Patient walk home after Injection.*—Even after simple tapping, I have several times known inflammation thus set up sufficient to bring about a radical cure; and I know of several cases where the patient has walked after injection, where great sloughing, and in one case death, ensued. I have notes of a case here where acute inflammation was thus set up, which terminated in gangrene and death in thirty-six hours, though the sac was not injected. William Knowlson, aged 64, was tapped in the surgery by one of the dressers, and about twelve ounces of common serum evacuated. Injection was not practised. He walked about four miles on the same day, and in the night was attacked with acute inflammation of the tunica vaginalis. The next day he was seen by a parish surgeon, who treated him for strangulated hernia, and afterwards sent him into this hospital, when there was great swelling of the scrotum and prepuce, and much irritative fever. Active treatment was adopted, but in less than twelve hours symptoms of gangrene of two-thirds of the scrotum came on, when stimulating poultices were applied, and he took calomel and opium with wine, porter, quinine, and as much nutriment as possible; but he died within 36 hours from the first attack. Sir Astley Cooper also mentions cases where dangerous symptoms came on after the patient had been allowed to walk; and one among others, of a gentleman who went down to Birmingham in the mail soon after injection, and sloughing of the whole of the scrotum followed. He also gives two cases, where he thought suppuration was thus produced.

*Dangers of the Seton.*—Here, gentlemen, is one of the cases which have led me to abandon the use of the seton, and to recommend you to follow my example. A military gentleman, aged 52, had twice submitted to injection of the tunica vaginalis, but without success. I therefore determined to employ the seton, which I passed with a long needle, after having drawn off the fluid. On the evening of that day there was but little uneasiness, and the seton was left in. The next morning he was doing well; inflammation not beyond the desirable extent; but at the evening visit there was considerable pain and swelling, with great anxiety of countenance. The seton was withdrawn, and leeches and cold lotions applied to the part. A dose of calomel and opium was also given.

Aug. 20. Delirious all night. Tumor increased in size, dark-coloured and ecchymosed. Port-wine poultice; fomentations; calomel and opium.

Evening. Delirium continued; bowels open; pulse 130, very compressible; tongue dry; brandy and water; ammonia and opium.

21. Scrotum in a state of slough; had a better night, less delirium; pulse 100; tongue moist. From this time he slowly improved, and ultimately recovered.

I had another case, in Job's ward, of a patient who had had hydrocele frequently injected without success. I therefore passed the seton. Violent symptoms followed. Suppuration took place; I therefore laid the tunic open, and the man ultimately recovered, but with great risk.

*Tetanus after the Operation.*—I remember a gentleman who submitted to this operation under the care of Sir Astley Cooper; wine and water being the injection used, which did not at the time produce more than the usual degree of pain. In twenty-four hours after, however, the patient complained of uneasy sensations about his jaw, attended with difficulty in swallowing, and shortly afterwards positive tetanus supervened. Calomel and opium were administered in large doses; fomentations were applied to the scrotum, which did not appear inordinately inflamed; and the patient ultimately recovered, although at one period of the case seemed hopeless. It may be worthy of remark that this gentleman was a native of the South of Spain.

*Calculus projecting in the Loins.**—I once saw a case of this kind with Sir Astley Cooper. He took me over to Limehouse, to see a gentleman who had an abscess, which was supposed to depend on disease of the hip joint; and, on opening this abscess, he put his finger to the bottom of the wound, and, feeling something hard and rough, thought it was a piece of exfoliating bone, but, when he had removed it by the forceps, it proved to be a urinary calculus. Well, after this, by a patient investigation of the symptoms, he was enabled to trace their cause from its formation in the kidney to its passage along the ureter, and then, as it had not reached the bladder, it had evidently passed through the ureter by ulceration, and excited suppuration in the gluteal region.

*Advantages of Leek Tea.*—I remember once going to Lambeth with Mr. Cline to see a patient; and this man had tied a cord across two of his bedposts, over which he threw his legs, and thus suspended himself by his hams. There was no doubt some ulceration of the bladder, and by this posture he made the stone gravitate from the ulcerated part. You may be inclined to say, when you consider what an excessively painful posture it must be to hang by hours together on a cord by the hams, that the remedy must be worse than the disease; but

---

* Prov. Med. and Surg. Journal, Feb. 13.

you must remember that pain is a comparative state; and it shows you what intense agony a man must suffer to lose the sense of pain, which must follow such a position, in the relief it afforded him. This man had refused an operation till it was too late, until, indeed, it was plain the bladder was so ulcerated, that no surgeon would operate. Mr. Cline was talking over various remedies, proposing first one and then another, all of which, it appeared, had been tried, till at last he said he had seen great relief follow leek-tea. Well, this was given him, and afforded the most astonishing relief, so much so, that he was enabled to resume a recumbent posture. However, it soon lost its effect, and the poor fellow died, completely worn out. I have seen it tried several times since, sometimes with and sometimes without effect; and I would advise you to bear it in mind, as worthy of trial, though you cannot foretel in what cases it will afford relief and when it will not.

*Never operate unless you hear the Stone at the time.*—I would lay it down as an axiom, that you should never perform an operation for the removal of stone from the bladder, unless you can hear the sound of the steel against the stone at the time of the operation. Do not be satisfied with the sense of touch, without that of hearing also. I distinctly felt and heard the stone in the bladder of the man now in the hospital this morning; but if I could not do so in the theatre to-morrow, I should put off the operation for that simple reason. You don't know what has occurred. The stone may have become sacculated; it may have passed out of the bladder through the urethra, or by ulceration; and in all these cases an operation would be improper.

*Sir Astley cut a Patient for Stone in his Consulting Room.*—A child was once brought to Sir Astley Cooper, in whose urethra he distinctly felt a stone. He therefore told his man to hold the child on his knees, and separate the thighs, just as we tie them in lithotomy, and then made a cut through the perineum upon the stone. However, just as he was about to withdraw it with the forceps, it slipped back into the bladder. He determined not to leave off till he had effected his object, and accordingly carried on his incision into the bladder, and removed the stone with a pair of dressing forceps. He took his guinea, and the child was taken home in a hackney-coach, and did uncommonly well; and I suppose Sir Astley is the only man who ever performed the operation of lithotomy on a mere morning patient in his consulting room.

---

### NEW YORK HOSPITAL.

Report of Surgical Cases. By John Watson, M.D. one of the Surgeons to the Hospital.*

We select some of the more interesting of the cases reported.

1. *Varicocele relieved by removing a portion of the Integuments of the Scrotum.* John Pierce, aged 21, a seaman, admitted June 23, with a large varicocele on the left side. On the day after admission, as much of the integuments of the scrotum as could be drawn together by the fingers over the enlarged vessels, was firmly seized and held together between the branches of a long pair of forceps, and the part above the forceps was then removed by one stroke of the bistoury. The flap appeared to form about one-third of the whole integuments

---

* New York Journ. Med. and Surg. Oct. 1840.

of the scrotum; and the retraction of the remaining portion was so great immediately after the operation, as to leave the whole of the tunica vaginalis on the left side exposed, and to allow the edges afterwards to be drawn together by sutures only with the greatest difficulty.

For several days after the operation, the patient continued comfortable; and though no portion of the wound united by adhesion, yet it contracted very rapidly, and presented a healthy granulating surface. But on the 1st of July, he was seized with a severe chill, with nausea and vomiting, which was followed by febrile excitement, and finally by a severe attack of erysipelas, which involve the integuments of the scrotum, penis, and parts adjacent, and extended to the sub-cutaneous cellular tissue in which it proceeded to suppuration. One or two openings formed along the base and body of the penis, continued to discharge freely for several days. As the erysipelatous inflammation subsided, the original wound, as well as the ulcerated openings, gradually contracted; and by the 20th of July, they had entirely healed.

The patient left the hospital on the 4th of August. The cicatrix produced by the healing of the wound was sufficient to support the distended veins, and prevent them from protruding much below the external abdominal ring; but they had not become permanently contracted or consolidated. After the patient had been for some time in bed, the swelling would appear to be effaced; but after he had been walking about the house all day, the enlargement of the veins was still apparent. He was, however, permanently relieved of the dragging sensation and pains of which he complained before the operation.

2. *Operation for Inversion of the Toe-nail.*

In this case the disease was of long standing, and had resisted every form of treatment, short of extirpation of the nail itself. The soft parts on both sides of the nail of the great toe on the right foot were ulcerated and in a fungous condition, and so sore as to prevent the patient from using his foot. Palliative means were employed until the irritability was in some degree subdued, and on the 21st of January the whole nail was removed by a modification of Lisfranc's operation.

The edge of a straight bistoury was carried perpendicularly through the integuments behind the matrix of the nail down to the bone, then directed forwards and carried horizontally under the nail toward the extremity of the toe; thus removing the nail and its matrix at a single sweep. The wound healed by granulations, and the patient was discharged on the 10th of February.

3. *Fracture of the Patella badly united by ligamentous union; followed by diffuse Inflammation and Suppuration in the neighbourhood of the Knee-joint.*

John Harrison, seaman, of Massachusetts, aged 28, admitted on the 8th of December, 1839, with an acute attack of inflammation which soon resulted in a diffuse abscess in front and on each side of the right knee-joint.

About eighteen months previous he had a transverse fracture of the patella, for which he was confined, with the limb in a straight position, about ten weeks. At the end of this time the fragments were in apposition; but as he began to use the limb the ligamentous union gradually yielded until the two fragments of bone became separated to the distance of three inches and a half from each other. In this condition he remained, able to walk about with a stick until the present attack. The inflammation and suppuration, though profuse, did not involve the cavity of the joint.

The case was treated as an ordinary abscess, and the patient cured February 10th, 1840.

*Contraction of the Neck from Injuries.*

Dr. Watson alludes to three cases.

In one of these, an injury behind the neck had produced a degree of tension and permanent inclination of the head towards the right shoulder. This deformity came on gradually a few days after the injury, and persisted about two months. The patient was finally relieved by local depletion, counter-irritation, and passive motion.

In another case, a similar injury led to paralysis of the muscles on the back of the neck. The patient was unable to support his head erect, the chin falling towards the breast.

In the third case, tension of the neck, owing probably to inflammation extending along the sheath of the right sterno-mastoid muscle, followed an acute attack of tonsilitis. The deformity continued about two weeks after the inflammation had left the throat and then gradually subsided.

*Tetanus treated by Assafœtida.*

Dr. Watson used the assafœtida in five cases. Two terminated favourably. He would prefer administering it by the rectum.

*Opium mixed with Superacetate of Lead, in large dose, swallowed with impunity.*

A seaman under treatment for a carious ulcer near the lower part of the thigh was seized with slight erysipelatous inflammation around the surface of the sore, for which the house-surgeon prescribed an opiate lotion, to be made from thirty grains of opium, and sixteen grains of sugar of lead. The patient, mistaking the directions, swallowed these substances in their dry state, about 8 o'clock in the evening of January 26th. About midnight he began to feel unwell, and think he threw up a part of the powder; but he did not rest well during the night; no symptoms of narcotism followed the accident; on the following morning he was as comfortable as usual, and unaware that anything had gone amiss.

"Is it possible that the superacetate of lead, which is known to precipitate the extractive matter of vegetable infusions, could in his case, have had any effect as an antidote? The opium was good, and the patient had not been in the habit of using this substance.

An instance somewhat similar happened in the hospital in the summer of 1837. A patient swallowed, by mistake, a drachm of opium mixed with a scruple of the superacetate of lead. The accident was discovered in about half an hour; an emetic was administered, and no serious consequences ensued."

---

### SOUTH UNION WORKHOUSE.

### Epidemic Ophthalmia.

Dr. Lees gives an account of this in the Dublin Journal for March.

The ophthalmia commenced on the 4th of June last, in a part of the institution (separate from the main body of the building) allotted for the children, and capable of containing four hundred, situated in an open and salubrious place, having a southerly aspect, but which, from being over crowded, as well as from some other defects, has proved the most unhealthy part of our establishment, an epidemic of petechial fever having broke out there a short time previously.

"The weather at this time was very cold, with sharp winds; so that in the first few cases I considered it as simple acute ophthalmia, excited by particles of dust having been blown into the eye; however it spread so rapidly, and assumed such a decided catarrhal character, with a peculiar state of conjunctiva and pupil, that I was led to regard it as an epidemic disease, and in this opinion I was confirmed by Mr. Creighton, who immediately recognized the disease as one which they frequently suffered from while he had charge of the Foundling Hospital, and which he regarded as of a highly contagious character."

The following case displays the features of the epidemic :—

Anne Magill, æt. 11, says she went to bed perfectly well on last night, (June 22,) she awoke with a sensation of sand in her left eye; there is now profuse lachrymation, with great itching; no intolerance of light; the pupil is largely dilated, and very sensitive, the palpebral conjunctiva, semilunar fold, and caruncle are very fluid, villous, with slight stringy discharge on the lower lid, which comes away easily, but forms again in a few minutes; there is no pain, except at night, nor constitutional disturbance complained of; but the tongue is swollen, flabby, coated with yellow fur.

℞. Calomel iij., Rhei. gr. x. ij. H. fiat Bolus.

And wash the eye with tepid water.

Next day the conjunctiva covering sclerotic was greatly congested, vessels of bright red with slight ecchymosis, as if they had given way in some places, profuse lachrymation, with stringy, mucous discharge from both eyes. Dr. Lees dropped the undiluted liquor plumbi into the eyes; it gave no pain; it was continued on the third day, she was nearly well, and, with bark and magnesia, she was quite cured in a week.

There was generally but little or no constitutional disturbance, in some there was slight catarrhal fever. The disease was, however, very liable to relapse more than once, and then became always more serious and intractable, particularly in strumous subjects, in whom it invariably became pustular, with a ferretty condition of eye, and great intolerance of light. It was contagious, for almost every adult who was occupied about these children suffered from its effects; but in them it generally assumed a more serious aspect, being more painful, and attended by severe chemosis.

The Report exhibits the good effects of the undiluted liquor plumbi.

---

### SIR PATRICK DUN'S HOSPITAL.

### Dr. Lendrick's Clinical Report.*

*It is a good thing sometimes to give up Physick.*—" It has long been my opinion, that the efficacy of treatment, in many chronic cases of disease, is misunderstood; and I have ventured to broach a theory which I think is confirmed by several facts, while it proposes the advantage of accounting for some extraordinary occurrences, and I allude to the operation of medicine, in saturating the constitution; and not only not relieving, but aggravating disease *during* its exhibition; although, on its administration being relinquished, the beneficial effects begin to appear. That this takes place, with respect to the administration of mercury, in venereal and some other diseases, is unquestionable. I think the principle may be extended to numerous medicines, and cases. That the disease does not subside *spontaneously* is proved by the circumstance, that, while formerly left to the efforts of nature, it became aggravated. That medicine generally succeeds better, in chronic diseases, by being occasionally laid aside and resumed, than by continued perseverance in its use, is a fact well known.

If this principle, that remedial treatment sometimes develops its effects slowly and subsequently to, instead of during a course of medicine, should be established by further experience, many difficulties connected with medical practice will be accounted for. We shall cease to be surprised, that after the efforts of experienced physicians have disappointed expectation, some empirical practitioner or inert medicine should *seem* to effect the cure; and many of the feats

---

* Dublin Journal, March, 1841.

accomplished by homœopathists, or attributed to imagination, may thus admit of solution."

We have observed that patients are generally better on giving up physick that does not suit their case. When it *does* they had better go on with it. Dr. Lendrick is a wag and will understand this.

2. *There is something in Animal Magnetism.*—" Animal magnetism is by most of the medical profession considered to be an imposture. I am, however, convinced, that in some cases a nervous influence exists between operator and patient, at least at a few inches distance; this conclusion I formed from witnessing our hospital experiments made on the *male* patient, *behind,* and under circumstances that precluded any suspicion. The opinion that a nervous *aura* exists beyond the material structure of nerves, has been held by some of the first modern physiologists."

" Future investigations must determine the extent of the operation of animal magnetism, and how far it can be rendered a remedial agent. To assert, without examination of the facts, that the system *must* be false, is unworthy of men of science; the same mode of reasoning, if it deserve the name, might have been applied to most discoveries. I confess, however, that I have not had opportunity, either at Sir Patrick Dun's Hospital or elsewhere, of determining the question of animal magnetism further than that there is *something* in it."

What that *something* is, we think we can tell the Doctor—it is just—*humbug.*

3. *Moxa.*—" The utility of moxæ, and the preference to be afforded to them rather than other modes of forming issues, were confirmed during my recent attendance at Sir Patrick Dun's Hospital. In a case, that of Flood, admitted November 15th, labouring under what is termed ' morbus coxæ senilis,' the effect of a severe fall, with shortening of the limb and infirmity; and in those of Mrs. Dolan and Mrs. Carolan suffering from obstinate sciatic neuralgia, the beneficial operation of moxa was especially remarkable. I have often tried the caustic issue in similar cases, but without equally beneficial results. There is something in the action of *fire,* of which farriers and other humble practitioners (of whom the learned might sometimes take a useful lesson) are fully aware.

Moxæ are applied at Sir Patrick Dun's Hospital by soaking the lint, of which they are formed, either in a saturated solution of *chromate* of potass, or in a solution of acetate of lead of the strength of about a drachm to the ounce of water; either of the solutions answers the purpose, and the opinions of the pupils were divided as to their relative advantages. The great point to be attended to is, to leave the moxa long enough on the part; this is determined, not only by the dark and shrivelled appearance of the skin surrounding the base of the moxa, but also by the *diminution* of pain on the sensibility of the surface being destroyed by the full accomplishment of the burning process. A piece of lint, dipped in water of caustic ammonia, immediately applied, as recommended by Baron Larrey, much mitigates the pain consequent on the application of the moxa; I question whether, on the entire, the suffering is as great as that from a caustic issue; the benefit derived is generally so considerable, that however the application of the first moxa may be dreaded and objected to, that of the second is often practised at the solicitation of the patient."

It is clear that the Dr. is a fire-worshipper, if not a fire-eater. We never saw a patient who asked for the second moxa, but many who have run away from it.

*Nauseous Physick.*—" I have for some time endeavoured to render medicine palatable without being inefficacious. This object is easily effected in the case of pectoral medicines, on account of the mucilaginous and syrupy ingredients which generally enter into their composition; as to others, the nauseous flavour may, in general, be much lessened by administering them in the state of *effervescence.*

During the present medical session I made several trials at Sir Patrick Dun's Hospital, with the above object. Astringent medicines in diarrhœa can scarcely be used in the state of effervescence, on account of the flatulence thus produced.

The ordinary chalk mixture I have often found to disagree with the stomach, and it does not seem to be of much efficacy, except in some cases, as a counteracter of acidity; I accordingly substituted a mixture, formed of syrup of orange, tincture of kino, tincture of rhatany, and a small quantity of laudanum. This, which was sportively named by some of the pupils "syrupus mirabilis," was found very effectual in cases of diarrhœa; and the patients declared it to be cordial and invigorating.

The producing the opposite state, that of purging, otherwise than by disagreeable means, is not at all so easy a matter. Most purgative medicines are very unpalatable; or, if the concentrated principle, such as that of croton oil, scammony, elaterium, &c., be brought to bear, severe griping and sickness are produced, at least in some constitutions. I have administered croton oil successfully in many cases, not only by diffusing a minute quantity throughout purgative pills, but also by mixing it with spirit and mucilage, and exhibiting it in quarter drop doses, with milk, as directed by Dr. Conwell. On the entire I prefer, for general use, two aperient pills, administered every three or four hours, and, after each dose, a seidlitz draught: for the former any of the usual formulæ may be used, and I have found melampodium a very useful adjunct. Where patients have a dislike to pills, senna mixture may be administered in effervescence. In hospital practice enemata afford ready means of effecting our object, after the failure of medicine given by the mouth. Although the prejudice against that description of remedy long impeded its use in private practice, except in case of downright necessity, it can now be so readily self-administered, or used by domestic means, that patients rarely object to it. In one hospital case, the necessity of using purgative medicine was considerably superseded by allowing the patient preserved tamarind pulp as a refreshment. Combinations of this, manna, honey, &c., are frequently used in nursery practice. There is no doubt that many palatable drinks (like the French ptisans) might be made to answer the two-fold purpose of beverage, and of aperient medicine."

*Teetotalism—Small-beer versus Tea and Coffee.*—"A man named Keegan, about 40 years old, was admitted into Sir Patrick Dun's Hospital, labouring under symptoms resembling delirium tremens. He suffered lately from epileptic fits, and his state appeared to border on insanity. His illness had commenced some months since, shortly after relinquishing habits of intoxication, and joining the Teetotal Society. He displayed much incoherence during his sojourn in the hospital, and left it in a few days seemingly in a state of derangement.

The question seems to be undetermined, as to how far intemperate habits can safely be relinquished *at once*. The balance of evidence is certainly in favour of the attempt proving successful; although the above case, and that of Burns, with the reports of some of the continental lunatic asylums, might lead to a contrary inference. I confess, however, I do not see the grounds on which the advocates of abstinence from strong liquors rush into the opposite extreme, and recommend the consumption of tea and coffee, articles which have nearly done as much to undermine the health of one sex, as the use of strong liquor has impaired the health of the other. Besides their own injurious effects, they lead to the baneful practice of opium eating. What objection have teetotalists to good table-beer, a home-made article, much more wholesome, and cheaper than tea or coffee?"

Tee-totalism is certainly another of the humbugs of the day. There is, however, this excuse for it. Being got up for the uneducated classes, it *must* be extravagant to catch them. The fanaticism of intoxication is met by the teetotal fanaticism.

